# Strain-Modulated Photoelectric Responses from a Flexible α-In_2_Se_3_/3R MoS_2_ Heterojunction

**DOI:** 10.1007/s40820-020-00584-1

**Published:** 2021-02-15

**Authors:** Weifan Cai, Jingyuan Wang, Yongmin He, Sheng Liu, Qihua Xiong, Zheng Liu, Qing Zhang

**Affiliations:** 1 Center for Micro- and Nano-Electronics, School of Electrical and Electronic Engineering, Nanyang Technological University, Singapore, 639798 Singapore; 2 School of Materials Science and Engineering, Nanyang Technological University, Singapore, 639798 Singapore; 3 Division of Physics and Applied Physics, School of Physical and Mathematical Sciences, Nanyang Technological University, Singapore, 639798 Singapore

**Keywords:** α-In_2_Se_3_/3R MoS_2_ heterojunction, Flexible, Self-powered photodetector, Strain modulation, Piezoelectric charge

## Abstract

Semiconducting piezoelectric α-In_2_Se_3_ and 3R MoS_2_ have attracted tremendous attention due to their unique electronic properties. Artificial van der Waals (vdWs) heterostructures constructed with α-In_2_Se_3_ and 3R MoS_2_ flakes have shown promising applications in optoelectronics and photocatalysis. Here, we present the first flexible α-In_2_Se_3_/3R MoS_2_ vdWs p-n heterojunction devices for photodetection from the visible to near infrared region. These heterojunction devices exhibit an ultrahigh photoresponsivity of 2.9 × 10^3^ A W^−1^ and a substantial specific detectivity of 6.2 × 10^10^ Jones under a compressive strain of − 0.26%. The photocurrent can be increased by 64% under a tensile strain of + 0.35%, due to the heterojunction energy band modulation by piezoelectric polarization charges at the heterojunction interface. This work demonstrates a feasible approach to enhancement of α-In_2_Se_3_/3R MoS_2_ photoelectric response through an appropriate mechanical stimulus.

**Supplementary material:**

The online version contains supplementary material available at (10.1007/s40820-020-00584-1) contains supplementary material, which is available to authorized users.

## Highlights

- A self-powered α-In_2_Se_3_/3R MoS_2_ heterojunction is successfully developed and shows strong photo response to the visible and near infrared light.
- The heterojunction photodetector delivers an ultrahigh photoresponsivity of 2.9 × 10^3^ A W^−1^ and a substantial specific detectivity of 6.2 × 10^10^ Jones under a compressive strain of − 0.26%.
- This work demonstrates that the transport of photo generated carriers is clearly modulated by mechanical stimuli through the piezo-phototronic effect at the heterojunction interface.

**Supplementary material:**

The online version contains supplementary material available at (10.1007/s40820-020-00584-1) contains supplementary material, which is available to authorized users.

## Graphical abstract

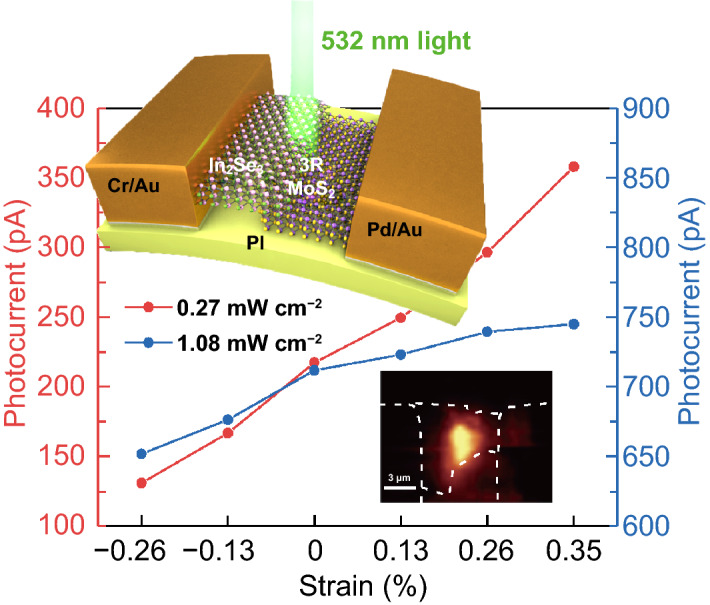

## Introduction

Since discovery of graphene, various two-dimensional (2D) materials, like hexagonal boron nitride, transition-metal dichalcogenides, oxides and chalcogenides, etc. have been successfully assembled into van der Waals (vdWs) heterostructures, uncovering their unique physical properties and developing novel electronic, optoelectronic, ferroelectric, thermoelectric and electrochemistry devices [1–4]. In addition, these 2D materials are of excellent mechanical properties which endow them a huge advantage in flexible electronic applications over conventional crystalline semiconductors which are very brittle. With appropriate stackings of these two-dimensional vdW materials, p–n junctions can form for the sake of development of flexible electronic and optoelectronic devices. Among these 2D materials, several ultrathin layers with non-centrosymmetric structure are of piezoelectricity and they are the most promising for mechanically modulated electronic and optoelectronic applications through mechanical agitations, like wurtzite structure material ZnO [5, 6]. Monolayer MoS_2_ has been employed to develop optoelectronic devices in which strain-induced piezoelectric polarization charges are utilized to modulate photoexcited carrier transport and recombination at the Schottky barrier or p–n junction interfaces. This strain-modulated process is called the piezo-phototronic effect [7–10]. However, the piezoelectricity of 2H MoS_2_ is restricted in odd few layers and it is significantly weakened with increasing the thickness [11]. In contrast, due to broken inversion symmetry, 3R MoS_2_ exhibits piezoelectricity from monolayer to the bulk, having an exciting potential for nonlinear optics, valley-dependent spin polarization, and advancing flexible wearable electronics [12, 13]. In addition, indium selenide, a direct bandgap and layered structure III–V compound, has recently attracted enormous attention, due to its superior electric, piezoelectric, thermoelectric, photoelectric and electrochemical properties [14–19]. Ding et al. theoretically revealed that In_2_Se_3_ and other III_2_-V_3_ van der Waals materials exhibit room-temperature ferroelectricity, originated from both spontaneous in-plane and out-of-plane electric polarization [20]. It is widely accepted that all ferroelectric materials are also piezoelectric [21, 22]. Indeed, the in-plane and out-plane ferroelectric and piezoelectric properties have been confirmed and characterized experimentally in α and β phase In_2_Se_3_ [23–26]. Theoretically speaking, controllable energy band alignment in a 3R MoS_2_ and In_2_Se_3_ heterostructure could be realized through an applied electric field, to achieve a broad spectrum of light absorption for novel tunable optoelectronic applications [27].

In this paper, we report on the first self-powered n-type α-In_2_Se_3_/p-type 3R MoS_2_ heterojunction photodetectors built on flexible substrate. These photodetectors show a good current rectification characteristic, ultrahigh photocurrent generation efficiency and highly sensitive photoresponse from the visible to near infrared region. The transport of photocarriers is strain-modulated at the heterojunction interface through the piezo-phototronic effect. With a + 0.35% tensile strain, the photocurrent can be enhanced by 64%, mainly promoted by piezoelectric polarization from In_2_Se_3_, and type-II band alignment between α-In_2_Se_3_ and 3R MoS_2_, which enhances the built-in electric field in the p-n heterojunction, in favor of photocarriers separation. To achieve high mechanical durability [28] and light absorption, the heterojunctions to be presented here were constructed with multilayer α-In_2_Se_3_ and 3R MoS_2_ flakes.

## Experimental Section

### Synthesis of α-In_2_Se_3_/3R MoS_2_ Heterojunction and the Device Fabrication

The device substrate was fabricated by spinning coating polyimide on a flexible polished stainless steel at 3000 rpm for 45 s, and then, annealed in argon gas at 250 °C for 2 h. The 3R MoS_2_ flakes were mechanically exfoliated from a chemical vapor deposition (CVD) synthesized bulk 3R MoS_2_ crystal onto the polyimide thin film. The surfaces of the MoS_2_ flakes were then treated with CHF_3_ plasma doping in a PlasmaThermo 790 MF Reactive ion etch (RIE) system. For the plasma doping, the RF power, gas pressure, precursor gas flow and process time were 100 W, 10 mTorr, 10 sccm, and 45 s, respectively. The bulk α-In_2_Se_3_ was bought on market and mechanically exfoliated onto the plasma treated 3R MoS_2_ flakes/polyimide. The overlapped α-In_2_Se_3_/3R MoS_2_ flakes were identified using optical microscopy. Cr/Au (10/150 nm) was deposited on the α-In_2_Se_3_ flakes and Pd/Au (10/150 nm) was coated on the 3R MoS_2_ flakes, using an e-beam evaporator and then patterned through a lift-off process.

### Materials Characterization

The atomic force microscopy (AFM) characterization (Cypher S Asylum Research Oxford Instruments) was carried out using non-contact mode. XPS measurements were conducted on a Kratos AXIS Supra X-ray photoelectron spectrometer.

The SHG measurement utilized a mode-locked Ti:sapphire laser (output wavelength: 800 nm and repetition rate: 76 MHz) to generate tunable wavelength light ranging from 500 to 1600 nm filtered through OPO, then circularly polarized by the quarter-wave plate, attenuated and focused on a sample by microscope objective lens (100 × , NA = 0.95). The SHG signal was collected by the same lens using a dichroic mirror and filtered by a short pass filter before entering a spectrometer.

The Raman scattering measurements (WITec alpha 300 confocal Raman microscopy) were carried out under a laser light of 532 nm, laser power of 0.1 mW and beam diameter of 400 nm with a 100 × objective lens.

### Electrical, Optoelectronic, and Mechanical Characterizations

The electrical characteristic measurements were performed using a Keysight B1500A Semiconductor Device Parameter Analyzer. 532 and 800 nm wavelength light with tunable intensity were obtained from a Quartz Halogen light system and a monochromator for the photovoltaic and photo-sensing measurement. The strains were applied through a home-made two-point bending apparatus. The strains applied were calculated through the bending angles (referring to the supporting document). For the spatial photocurrent mapping, the sample was fixed on the motorized stage in WITec alpha 300 confocal Raman microscopy with a continuous 532 nm laser with a beam diameter of 400 nm. The photocurrent was measured using Keithley 2450 sourcemeter, and a Femto DLPCA-200 universal low noise current amplifier.

## Results and Discussion

**Fig. 1:**
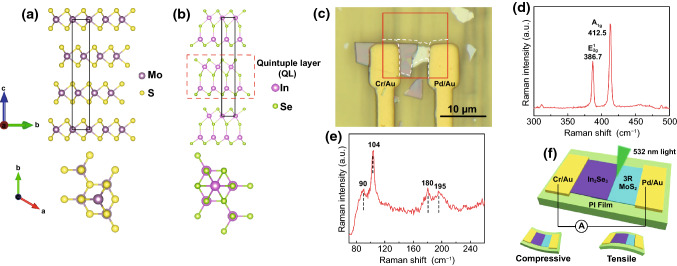
Atomic structures of 3R phase MoS_2_ and Hexagonal α-In_2_Se_3_, and optical, Raman spectra, electrical measurement setup of an α-In_2_Se_3_/3R MoS_2_ heterojunction. **a** Side and top view of 3R MoS_2_ atomic structure. The purple and yellow spheres correspond to molybdenum and sulfur atoms, respectively. **b** Side and top view of hexagonal α-In_2_Se_3_ atomic structure. The pink and green spheres correspond to indium and selenide atoms, respectively. **c** Optical image of the heterojunction on a flexible substrate. **d** Raman spectrum of the 3R MoS_2_ flake. **e** Raman spectrum of the α-In_2_Se_3_ flake under the excitation of 532 nm wavelength laser. **f** Schematic diagram of the heterojunction on a flexible substrate

3R MoS_2_ is of a broken symmetry, regardless of the layer number, by repeating ABC-ABC stacking order, where A, B, C are three same monolayer MoS_2_ in the same direction with a shift, as shown in Fig. 1a for the side and top views. Therefore, 3R MoS_2_ is piezoelectric and its piezoelectric coefficient *e*_11_ is theoretically calculated to be around 0.40 C m^−2^ for 1–6 layers and 0.30 C m^−2^ for the bulk [13]. Our recent piezoelectric force microscopy (PFM) measurements suggest an out-of-plane piezoelectric coefficient *d*_33_ of 1.2 pm V^−1^ for a 28 nm thick 3R MoS_2_ flake [29]. α-In_2_Se_3_ typically has hexagonal or rhombohedral atomic structures. Both structures have primary quintuple layers in different stacking orders. A single quintuple layer consists of five alternately arranged Se–In–Se–In–Se atomic layers, as illustrated in Fig. 1b. Hexagonal α-In_2_Se_3_ at any thickness possesses non-centrosymmetric property along the vertical direction, leading to the out-of-plane piezoelectricity (*d*_33_). In a single quintuple layer, one In atom and two Se atoms are located at nonequivalent sites of the hexagonal structure, generating the in-plane (*d*_11_) piezoelectricity under a planar strain. Recent theoretical calculation suggests a higher magnitude d_13_ than that of d_33_, implying that a significant vertical piezoelectric polarization could be induced under an in-plane strain. Bilayer hexagonal α-In_2_Se_3_ constructed by two dislocated quintuple layers results in the reservation of non-centrosymmetry, so does a multilayer hexagonal α-In_2_Se_3_ flake. Thus, hexagonal α-In_2_Se_3_ is of in-plane and out-of-plane piezoelectricity at any thickness [20, 23, 24, 30].

To prepare a heterojunction of 3R MoS_2_ & α-In_2_Se_3_ flakes, the flakes were mechanically exfoliated from 3R MoS_2_ and α-In_2_Se_3_ crystals and then deposited on a clean flexible polyimide thin film in sequence. As shown in Fig. 1c, a location with a 3R MoS_2_ flake overlapped with an α-In_2_Se_3_ flake was selected through an optical microscope. Two characteristic Raman peaks shown in Fig. 1d, in-plane mode (${E}_{2g}^{1}$) and out-of-plane mode (${A}_{1g}$), were observed from the bottom 3R MoS_2_ flake and the polarization-resolved second-harmonic generation (SHG) measurement evidenced a non-centrosymmetric structure from sixfold pattern (see Fig. S1), confirming that the bottom flake was indeed a 3R MoS_2_ flake. Four characteristic Raman peaks at 90, 104, 180, and 195 cm^−1^ from the top flake (shown in Fig. 1e) indicate a hexagonal structure of an α-In_2_Se_3_ flake. Furthermore, the bottom 3R MoS_2_ flake was p-type semiconducting after CHF_3_ plasma treatment and the top α-In_2_Se_3_ was n-type, as characterized by the x-ray photoelectron spectroscopy (XPS) measurement described in Fig. S1 [31–34]. A Cr/Au (10/150 nm) electrode and a Pd/Au (10/150 nm) electrode were deposited on the α-In_2_Se and 3R MoS_2_ flakes, respectively, to achieve ohmic contacts as explained in Fig. S2. The device and the circuit connection are illustrated in Fig. 1f. To study the strain modulation on the p–n heterojunction, the uniaxial compressive and tensile strains were applied through bending the flexible device downward and upward, see the insets in Fig. 1f. The strains applied were calculated with the bending angles as discussed in Fig. S3. The flexible heterojunction was characterized by a semiconductor parameter analyzer (Agilent B1500A) under different wavelength illumination and intensities from a Quartz Halogen light system through a monochromator. The morphology and height profiles of the heterojunction showed that the thickness of the 3R MoS_2_ and α-In_2_Se_3_ flakes was 30 and 206 nm, respectively (Fig. S4).

**Fig. 2:**
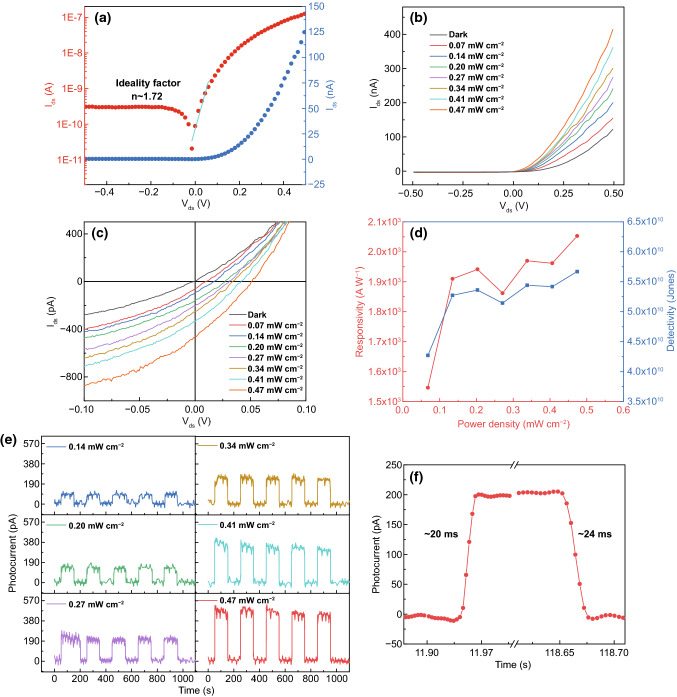
Electrical characterization and photoresponse from the α-In_2_Se_3_/3R MoS_2_ heterojunction under zero strain. **a** *I*–*V* characteristic in the dark with the logarithmic and linear scale. **b, c** *I*–*V* characteristics in the dark and under illumination of 532 nm wavelength under different light intensities from − 0.5 to 0.5 and − 0.1 to 0.1 V, respectively. **d** Responsivity and detectivity at a bias voltage of 0.5 V as a function of illumination intensities. **e** Current vs time under 532 nm illumination with several intensities and zero bias voltage. **f** Current vs time extracted from **e** under the illumination intensity of 0.27 mW cm^−2^

The dark *I-V* characteristic from the heterojunction device, as shown in Fig. 2a, was measured under the strain-free condition. The ideality factor was found to be 1.72 and the rectification factor under bias voltages of ± 0.5 V was 405. The observed excellent rectification characteristic indicates formation of a high-quality p–n junction. Under 532 nm light illumination with an intensity of 0.47 mW cm^−2^ and a bias voltage of + 0.5 V, a fourfold rise in the current, *I*_ds_, from 123 nA in the dark to 415 nA was observed, see Fig. 2b. The *I*–*V* characteristics measured under the intensity from 0.07 to 0.47 mW cm^−2^ and bias voltage of ± 0.1 V are shown in Fig. 2c. The photoresponsivity $= \frac{{\mathrm{I}}_{\mathrm{light}}-{I}_{\mathrm{dark}}}{{P}_{t}*A}$, (where *P*_*t*_ and *A* are the illumination intensity and effective area of the heterojunction), was found to be 2.1 × 10^3^ A W^−1^ at an intensity of 0.47 mW cm^−2^. Compared with those 2D materials-based photodetectors reported by other groups, especially the heterojunction devices listed in Table 1, our p–n heterojunction device has showed an ultrahigh photoresponsivity at a very low bias voltage, owing to a higher photocurrent generation efficiency and lower power consumption [35, 36]. The specific detectivity,${ D}^{*}= \frac{R}{\sqrt{\frac{2q{I}_{\mathrm{dark}}}{A}}}$, (where *R* and *q* are the responsivity and elementary charge), is usually used to tell the capability of detecting the incident photons. The D* value for our device was about 5.7 × 10^10^ Jones at the intensity of 0.47 mW cm^−2^, showing a good sensitivity to the light. The *R* and D* under several illumination intensities and a bias voltage of + 0.5 V are given in Fig. 2d. Under zero bias voltage and the light intensity of 0.27 mW cm^−2^, the *R* and D* were 2.5 A W^−1^ and 2.1 × 10^10^ Jones, respectively. Therefore, this α-In_2_Se_3_/3R MoS_2_ heterojunction could function as a self-powered photodetector. The photocurrent could stabilize from 100 to 500 pA under various light intensities with the rise and fall time of 20 and 24 ms shown in Fig. 2e, f. This device demonstrated a faster light response than most of the In_2_Se_3_ based photodetectors developed by other groups [18, 37–40]. Recent theoretical calculation predicts that the photocurrent generation from a α-In_2_Se_3_/3R MoS_2_ heterojunction could cover from visible light to near infrared region, with a higher optical absorption coefficient and current density than an isolated In_2_Se_3_ layer [27]. Indeed, infrared photoresponse from few layer β-In_2_Se_3_/monolayer MoS_2_ heterojunctions showed an extended detection range from the visible to near infrared region [37], due to the relatively small bandgap of β-In_2_Se_3_. As few layer β-In_2_Se_3_ and monolayer MoS_2_ were involved, the light absorption of their devices could not be high. The photoresponse from our α-In_2_Se_3_/3R MoS_2_ heterojunction devices did cover the visible and near infrared regions, as shown in Fig. S5. Compared with their results, our device showed superior performance with three orders higher responsivity and detectivity. It is easily found from Fig. S5c, e that the photocurrent under 532 nm illumination was around four times larger than that under 800 nm illumination, very likely due to weak light absorption in the near infrared region [41].

**Table 1:** Comparison of the characteristic parameters of our present heterojunction devices with other 2D materials-based photo devices

Materials	*λ* (nm)	*V*_bias_ (V)	Responsivity (A W^−1^)	Detectivity (D*)	Response time (ms)	Refs
α-In_2_Se_3_/3R MoS_2_	532	0.5	2052	5.7 × 10^10^	20	This work
MoS_2_ Monolayer	442	2	2.3 × 10^4^	NA	NA	[7]
MoS_2_ Monolayer	532	− 10	1162	1.7 × 10^12^	NA	[8]
MoS_2_ Multilayer	532	1.2	59	NA	0.042	[35]
α-In_2_Se_3_ Monolayer	532	2	340	NA	6	[15]
α-In_2_Se_3_ Nanosheets	300	5	395	2.3 × 10^12^	18	[16]
In_2_Se_3_ nanosheets	532	5	20.5	6.0 × 10^11^	24.6	[17]
β-In_2_Se_3_ flake	650	20	3.8	1 × 10^10^	3870	[18]
WSe_2_/CdS	680	2	33.4	NA	NA	[36]
MoS_2_ /WSe_2_	532	NA	1.8 × 10^−3^	NA	NA	[9]
β-In_2_Se_3_ /MoS_2_	450	1	4.47	1.1 × 10^9^	52	[37]
MoS_2_ /GaN	365/685	20	127 @ 365 33 @ 685	1.1 × 10^11^	1500 @ 365 8000 @ 685	[38]
β-In_2_Se_3_ /GaN	365/685	20	1.6 @ 365 0.3 @ 685	1.6 × 10^9^	210 @ 365 2300 @ 685	[38]
CuO/MoS_2_	432	10	NA	3.3 × 10^8^	NA	[10]
WSe_2_/α-In_2_Se_3_	650	− 1	0.026	NA	2.3	[39]
GaN/α-In_2_Se_3_	365/850	3	127 @ 365 33 @ 850	3.6 × 10^10^ @ 850	130 @ 365 200 @ 850	[40]

**Fig. 3:**
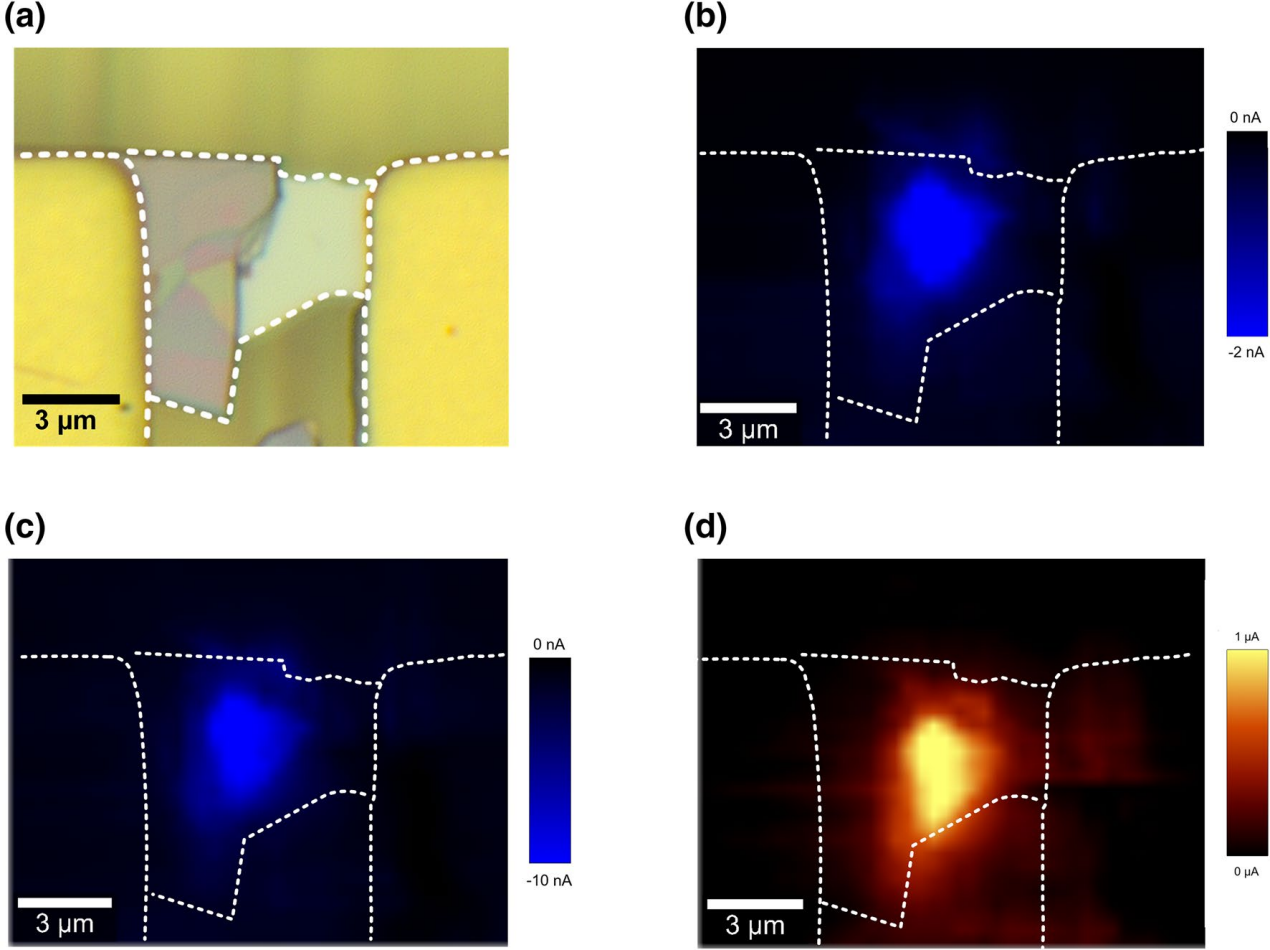
Optical scanning photocurrent images of the α-In_2_Se_3_/3R MoS_2_ heterojunction. **a** The optical image. **b**–**d** Photocurrent mapping of the heterojunction under zero bias, reverse bias of − 0.25 V and forward bias of 0.25 V at 532 nm with a laser power of 0.1 mW and a spot waist radius of 400 nm

To tell that the photoresponse was dominated by the heterojunction, rather than from the α-In_2_Se_3_ or 3R MoS_2_ flake, a scanning photocurrent microscopic image (SPCM) was performed on the entire area between the two electrodes using a WITec Raman system with laser light of 532 nm at a power of 0.1 mW (See Experimental section for experimental setup). The SPCM images shown in Fig. 3 clearly displayed that it was only in the heterojunction area where the photocurrent was apparently generated when the α-In_2_Se_3_/3R MoS_2_ heterojunction was applied with a reverse bias of − 0.25 V, zero bias and a forward bias of 0.25 V.

**Fig. 4:**
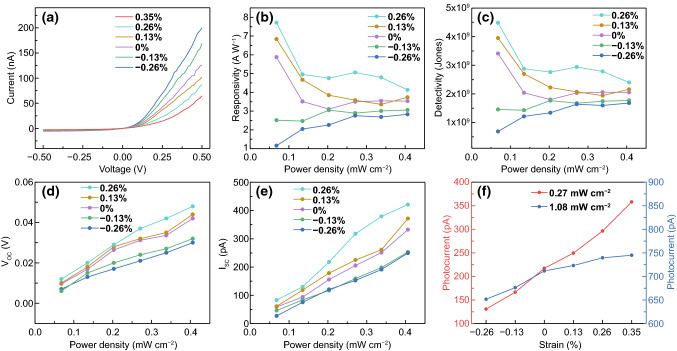
The strain-modulated photoresponse to 523 nm illumination from the α-In_2_Se_3_/3R MoS_2_ heterojunction. **a** *I*–*V* characteristic of the device under several strains in dark. **b**,** c** Responsivity and detectivity under − 0.1 V bias voltage. **d**,** e** Open circuit voltage *V*_oc_ and short circuit current *I*_*s*c_ under various light intensities and strains (The data were extracted from Fig. S7). **f** Average photocurrent as a function of strains under zero bias at the illumination intensities of 0.27 and 1.08 mW cm^−2^ (The data were extracted from Fig. S8)

To study the influence of strains on photoresponse, the photocurrent was measured under different strains and light intensities. The *I*–*V* curves under various strains in dark are displayed in Fig. 4a. Apparent strain-modulated *I*–*V* characteristics can be seen with enhancing (weakening) rectification characteristics under compressive (tensile) strains. With a bias voltage of + 0.5 V, the current *I*_ds_ was increased from 136 to 200 pA under a compressive strain of − 0.26% and decreased to 65 pA under a tensile strain of + 0.35%. Upon a compressive strain of − 0.26%, the responsivity increased from 1.5 × 10^3^ to 2.9 × 10^3^ A W^−1^, by 88%, while the detectivity rose from 4.3 × 10^10^ Jones to 6.2 × 10^10^ Jones, by 46% (Fig. S6e, f). Under a bias voltage of − 0.1 V, the strain modulations of the responsivity and detectivity occurred at a low illumination intensity of 0.07 mW cm^−2^ were much more significant than at a high intensity of 0.41 mW cm^−2^, as shown in Fig. 4b, c. The responsivity and detectivity decreased by 80% (from 5.9 to 1.2 A W^−1^) and 80% (3.4 × 10^9^ to 6.9 × 10^8^ Jones), under a compressive strain of − 0.26%. The open circuit voltage *V*_oc_ and short circuit current *I*_sc_ were increased with increasing the tensile strain but decreased with increasing the compressive strain as shown in Fig. 4d, e. In Fig. 4f, under a low light intensity of 0.27 mW cm^−2^, the photocurrent was increased from 220 to 360 pA (by 64%) up on a tensile strain of + 0.35%, but decreased from 220 to 130 pA (by 41%) with a compressive strain of − 0.26%. In contrast, under a high illumination intensity of 1.08 mW cm^−2^, it was only increased from 710 to 750 pA (by 5.6%) under the tensile strain and decreased from 710 to 650 pA (by 8.5%) under the compressive strain. The photocurrents as a function of time under 0.27 mW/cm^2^ and 1.08 mW cm^−2^ were also shown in Fig. S8. Illumination intensity dependent strain modulation of the photocurrent may not be accounted for by the strain-induced optical light absorption coefficient change in the 2D vdWs flakes, as the theoretical calculation has indicated that only a small change in light absorption coefficient in a 2% strained In_2_Se_3_/MoS_2_ heterojunction from ultraviolet to near-infrared light range [41]. As the strains applied in this study were much smaller than 2%, the strain-induced optical light absorption coefficient change in the 2D flakes can be ignored. In Fig. S2, the In_2_Se_3_ and MoS_2_ devices with good ohmic contacts did not show detectable electrical transport modulation under mechanical strains. These phenomena rule out the contribution from piezoresistive effect. Instead, the findings could be well interpreted using a piezoelectric potential originated from the piezoelectric charges at the heterojunction interface.

**Fig. 5:**
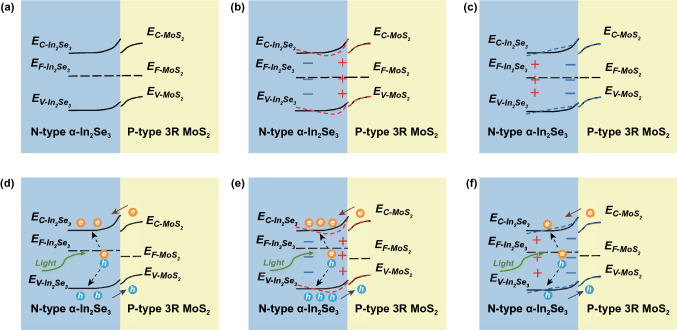
The energy band diagrams for the α-In_2_Se_3_/3R MoS_2_ heterojunction with\without the strains and light illumination. The energy band diagram **a** in thermal equilibrium with zero light illumination and no external strain **b** under zero light illumination, a tensile strain **c** under zero light illumination, a compressive strain **d** under light illumination, no strain applied **e** under light illumination, a tensile strain **f** under light illumination and a compressive strain

To interpret the strain-modulated photoresponse, the energy band diagrams of the heterojunction are plotted in Fig. 5. When a 3R MoS_2_ flake (with an indirect band gap of 1.29 eV and a higher electron affinity of 4.0 eV) is in contact with an α-In_2_Se_3_ flake (having a direct band gap of 1.55 eV and a lower electron affinity of 3.6 eV) [31, 41–45], a negative (positive) space charge region in the 3R MoS_2_ (α-In_2_Se_3_) flake is established, forming a p-n heterojunction in the thermal equilibrium under zero strain. The widths of the depletion region located in 3R MoS_2_ and α-In_2_Se_3_ sides can be estimated using the depletion model for a conventional p–n heterostructure, i.e. ${x}_{p}=\sqrt{\frac{2{N}_{d}{\varepsilon }_{{In}_{2}{Se}_{3}}{\varepsilon }_{{MoS}_{2}}{V}_{bi}}{q{N}_{a}({\varepsilon }_{{In}_{2}{Se}_{3}}{N}_{a}+{\varepsilon }_{{MoS}_{2}}{N}_{d})}}$ and ${x}_{n}=\sqrt{\frac{2{N}_{a}{\varepsilon }_{{In}_{2}{Se}_{3}}{\varepsilon }_{{MoS}_{2}}{V}_{bi}}{q{N}_{d}({\varepsilon }_{{In}_{2}{Se}_{3}}{N}_{a}+{\varepsilon }_{{MoS}_{2}}{N}_{d})}}$, where ${N}_{a}$ and ${N}_{d}$ are the doping concentrations in the 3R MoS_2_ and α-In_2_Se_3_ flakes, respectively; ${V}_{bi}$ is the built-in voltage; *q* is the elementary charge, ${\varepsilon }_{{MoS}_{2}}$ and ${\varepsilon }_{{In}_{2}{Se}_{3}}$ are the permittivity of 3R MoS_2_ and α-In_2_Se_3_ flakes, respectively [46–49]. As the carrier concentration, layer thickness and permittivity for the MoS_2_ (In_2_Se_3_) flake are $p \approx 2.3 \times 10^{12} \left( {n \approx 1.2 \times 10^{12} } \right)$ cm^−3^, ${t}_{{\mathrm{MoS}}_{2}}\approx 30 ({t}_{{\mathrm{In}}_{2}{\mathrm{Se}}_{3}}\approx 206)$ nm, ${\varepsilon }_{{MoS}_{2}}=6.9\times {\varepsilon }_{0}$(${\varepsilon }_{{In}_{2}{Se}_{3}}=17\times {\varepsilon }_{0}$) [50–53]. where ${\varepsilon }_{0}$ is the permittivity in vacuum, the doping concentration could be estimated as ${N}_{a}=\frac{n }{{t}_{{\mathrm{In}}_{2}{\mathrm{Se}}_{3}}}\approx 5.7\times {10}^{16} ({N}_{d}=\frac{p }{{t}_{{\mathrm{MoS}}_{2}}}\approx 7.7\times {10}^{17})$ cm^−3^. The built-in voltage ${V}_{bi}\approx \frac{KT}{q}\mathrm{ln}\left(\frac{{N}_{a}{N}_{d}}{np}\right)\approx 0.61$ V, for the type II band alignment, and the depletion width in the 3R MoS_2_ (α-In_2_Se_3_) flake should be ${x}_{p}\approx 130$ (${x}_{n}\approx 9.7$) nm. As the depletion width in the MoS_2_ flake exceeded the thickness of MoS_2_ flake, the 3R MoS_2_ could be fully depleted. Since the theoretical d_13_ magnitude of α-In_2_Se_3_ is 3.08 pm V^−1^, a factor of 15 larger than that of 3R MoS_2_, we could ignore the out-of plane piezoelectric polarization in the 3R MoS_2_ flake for simplicity of discussion [30, 54]. Under a tensile (compressive) strain in dark, positive (negative) piezoelectric charges could emerge at the bottom surface of the α-In_2_Se_3_ flake [50]. The positive (negative) piezoelectric charges lower (raise) the energy band near the interface of the α-In_2_Se_3_ flake, as shown in Fig. 5b, c. The total internal electric field is enhanced (weakened) so that the potential barrier height is increased (decreased) in comparison with the condition of zero strain. As a result, the current under a forward bias would be reduced (enhanced) so that the rectification factor was found to be smaller (larger) under a tensile (compressive) strain, as shown in Fig. 4a. Upon illumination, only those electron–hole pairs created in or near the heterojunction could be separated by the built-in electric field with electrons (holes) being swept into α-In_2_Se_3_ (3R MoS_2_), giving rise to a photocurrent, see Fig. 5d. Upon a tensile (compressive) strain applied, the total internal electric field is enhanced (weakened) and the electric potential difference is then increased (decreased) in the heterojunction as discussed above, in favor of (weakening) separation of the electron–hole pairs and injection efficiency in or near the heterojunction (Fig. 5e, f), causing *V*_oc_ and *I*_sc_ increasing (decreasing) with raising tensile (compressive) strain, as shown in Fig. 4d, e. At a high illumination intensity, the number of electron hole pairs in or near the heterojunction is greatly increased so that the piezoelectric polarization charges could be effectively screened. This could equivalently reduce strain modulation effect [7–9], in consistence with our experimental finding of the light intensity dependence of the photocurrent modulation in Fig. 4.

## Conclusion

High performance flexible heterojunction photodetectors have been successfully developed by stacking an α-In_2_Se_3_ flake with a 3R MoS_2_ flake. The devices showed clear photocurrent response to visible and near infrared light. The photocurrent response was found to be enhanced (reduced) with a tensile (compressive) strain and the strain modulation of the photocurrent response was much more significantly under weak illumination than under strong illumination. The strain modulation can be interpreted from the strain-induced piezoelectric polarization charges, which alter the total internal electric field in the heterojunction, promoting (weakening) collection of the photocarriers.

## Electronic supplementary material

Below is the link to the electronic supplementary material.

Supplementary material 1 (PDF 712 kb)
