# Supplementary material for: Strain-Modulated Photoelectric Responses from a Flexible α-In2Se3/3R MoS2 Heterojunction
Source: Nanomicro Lett. 2021 Feb 15;13:74. doi: 10.1007/s40820-020-00584-1 (PMC8128968; doi:10.1007/s40820-020-00584-1)
Supplement: Supplementary file 1 — Supplementary material 1 (PDF 712 kb) [file 40820_2020_584_MOESM1_ESM.pdf]

Supporting Information for

Strain Modulated Photoelectric Responses from a Flexible α-In_2Se_3/3R MoS_2 Heterojunction

Weifan Cai[1], Jingyuan Wang[1], Yongmin He[2], Sheng Liu[3], Qihua Xiong[3], Zheng Liu[2], Qing Zhang[1, *]

[1]Centre for Micro- & Nano-Electronics, School of Electrical and Electronic Engineering, Nanyang Technological University, Singapore 639798, Singapore

[2]School of Materials Science and Engineering, Nanyang Technological University, Singapore 639798, Singapore

[3]Division of Physics and Applied Physics, School of Physical and Mathematical Sciences, Nanyang Technological University, Singapore 639798, Singapore

*Corresponding author. E-mail: eqzhang@ntu.edu.sg (Qing Zhang)

Supplementary Tables and Figures

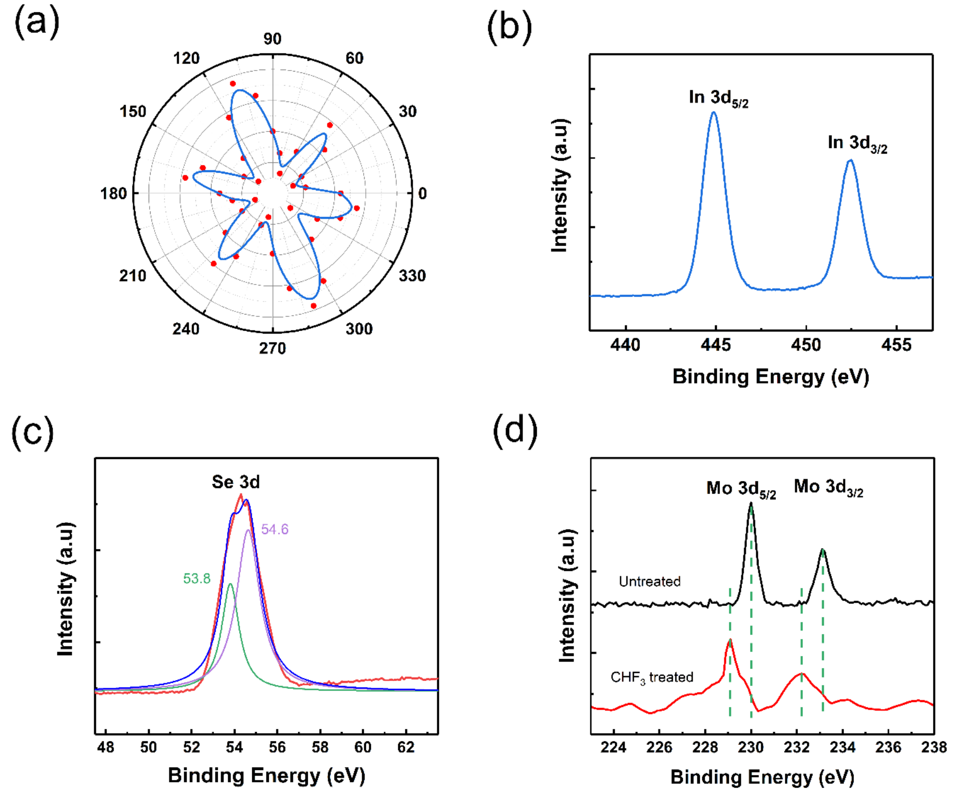

Fig. S1 a Polarization dependence plot of SHG intensity of 3R MoS_2 flake in the heterojunction **(Fig. 1c**). The red dots are experimental data, and the blue solid lines are fitting lines. **b, c** XPS spectra of In *3d* and Se *3d* core orbital peaks from the α-In_2Se_3 flake. **d** Mo *3d* core orbital peaks from the 3R MoS_2 flake before and after CHF_3 plasma treatment

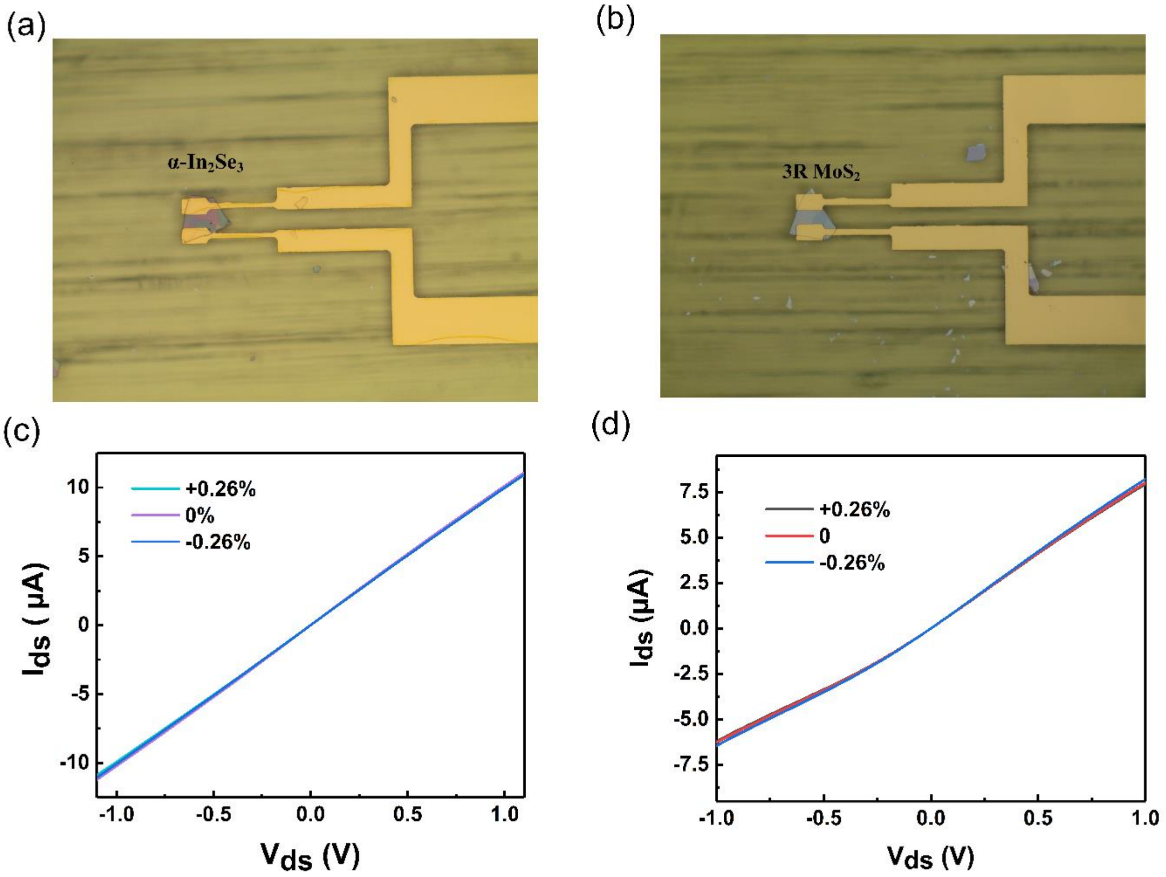

Fig. S2 Optical images and *I-V* characteristics of α-In_2Se_3 and 3R MoS_2 flakes. **a** One pair of Cr/Au (10/150nm) electrodes were deposited on an α-In_2Se_3 flake. **b** One pair of Pd/Au (10/150nm) electrodes were deposited on a 3R MoS_2 flake. **c** *I-V* characteristics of the α-In_2Se_3 sample shown in **a** with no strain, a tensile strain of +0.26% and a compressive strain of -0.26%. **d** *I-V* characteristics of the 3R MoS_2 sample shown in **b** with no strain, a tensile strain of +0.26% and a compressive strain of -0.26%

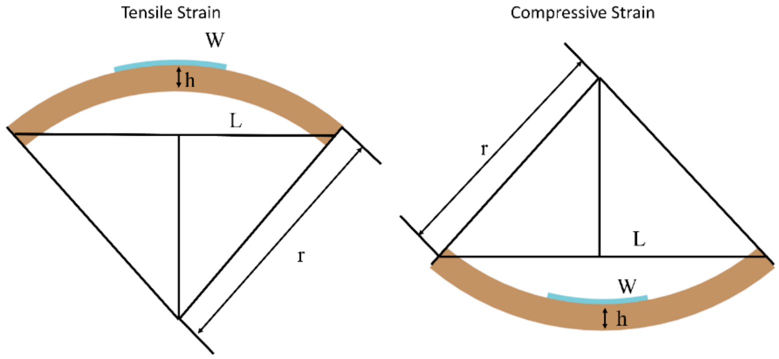

Fig. S3 Illustration of the mechanical strain applied onto the thin layers in blue

Since the dimensions of the α-In_2Se_3 and 3R MoS_2 flakes (~20 μm long and a few tens of nanometer thick) are much smaller than the PI substrate deposited on the stainless steel (15 mm × 15mm × 300 μm), the tensile and compressive strains exerted onto the flakes deposited onto

the PI substrate could be given by ε = h/2r, where h is the thickness of the PI substrate, L is the bended length of the device and r is the bending radius. The strain ε was calculated and shown in Table S1.

Table S1 Parameters for calculating the applied strains

H (μm)	L (mm)	r (mm)	ε
300	14	11.74	0.13%
300	11.5	5.68	0.26%
300	8.5	4.31	0.35%

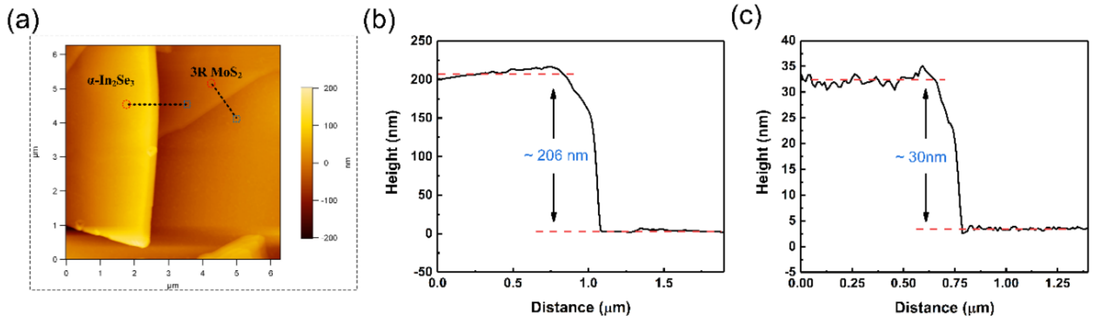

Fig. S4 a AFM image of the α-In_2Se_3/3R MoS_2 heterojunction (**Fig. 1c**). **b** Height profile of the α-In_2Se_3 flake. **c** Height profile of the 3R MoS_2 flake

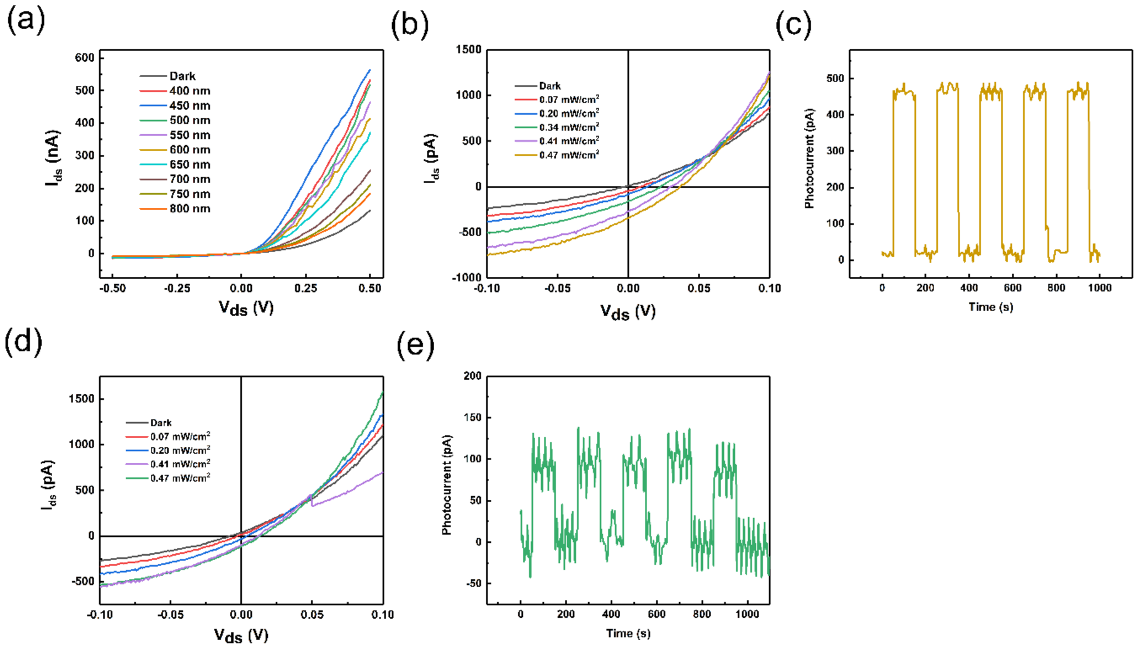

Fig. S5 Photoresponse from the α-In_2Se_3/3R MoS_2 heterojunction (Fig. 1c) under several different light intensities under zero strain. **a** *I-V* characteristic under illumination intensity of 0.47 mW/cm^2 with different wavelengths. **b** *I-V* characteristics under the dark and 532 nm illumination with different intensities. **c** Current vs time under the 532 nm illumination intensity of 0.47 mW/cm^2. **d** *I-V* characteristics under the dark and 800 nm illumination with different intensities. **e** Current vs time under 800nm illumination intensity of 0.47 mW/cm^2 with zero bias voltage

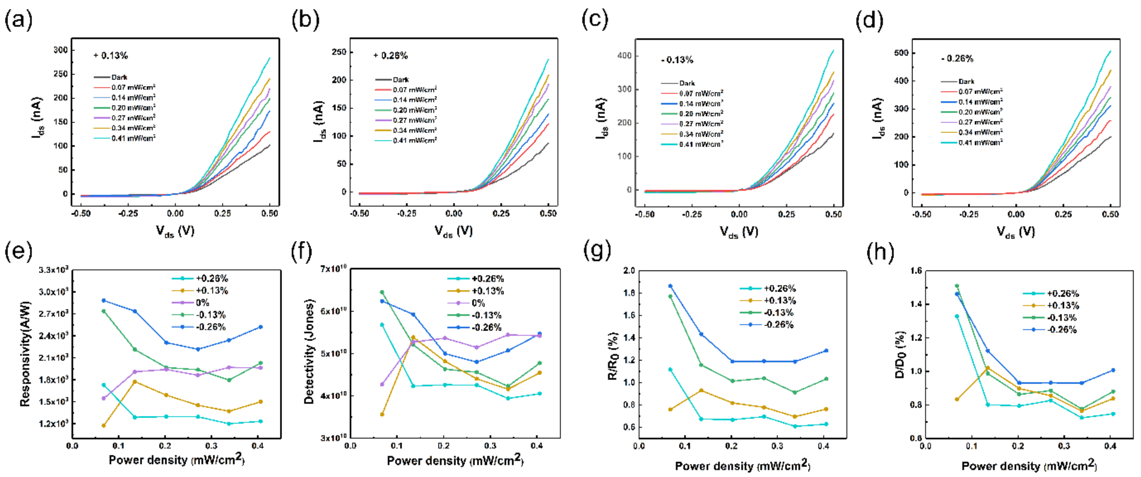

Fig. S6 a-d *I-V* characteristics of the α-In_2Se_3/3R MoS_2 heterojunction (Fig 1c) from -0.5 V to 0.5 V under the dark and various 532 nm light illumination intensities with the strain of +0.13%, +0.26%, -0.13% and -0.26%. **e-f** Responsivity, detectivity, relative change of the responsivity and relative change of detectivity with respect to that under zero strain as a function of the light intensities and strains under +0.5 V bias (The data were extracted from **Fig. S6a-d**)

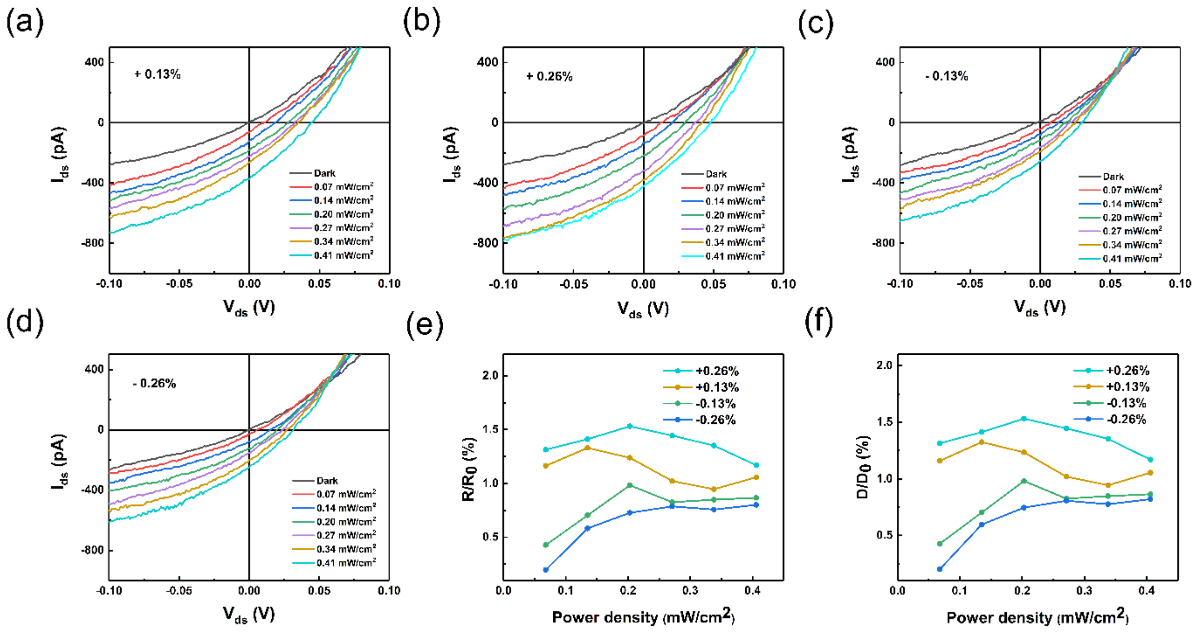

Fig. S7 a-d *I-V* characteristics of the α-In_2Se_3/3R MoS_2 heterojunction (**Fig. 1c**) from -0.1 V to 0.1 V under the dark and illumination of 532 nm wavelength under different light intensities with the strain of +0.13%, +0.26%, -0.13% and -0.26%. **e, f** Relative change of the responsivity and detectivity with respect to that under zero strain as a function of the light intensities and strains under the bias voltage of -0.1 V (The data were extracted from **Fig. S7a-d**)

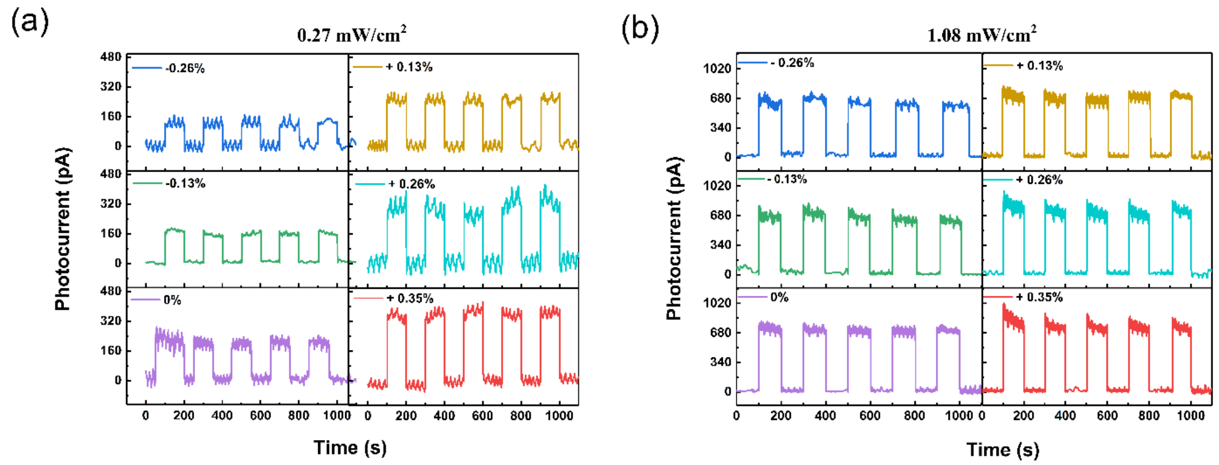

Fig. S8 a, b *I*-*t* of the heterojunction (**Fig. 1c**) under zero bias and several strains at the 532 nm light intensity of 0.27 mW/cm^2 and 1.08 mW cm^{-2}
